# Supplementary figures and images for: Survival benefits of perioperative chemoradiotherapy versus chemotherapy for advanced stage gastric cancer based on directed acyclic graphs
Source: PLoS One. 2023 Apr 14;18(4):e0283854. doi: 10.1371/journal.pone.0283854 (PMC10104374; doi:10.1371/journal.pone.0283854)

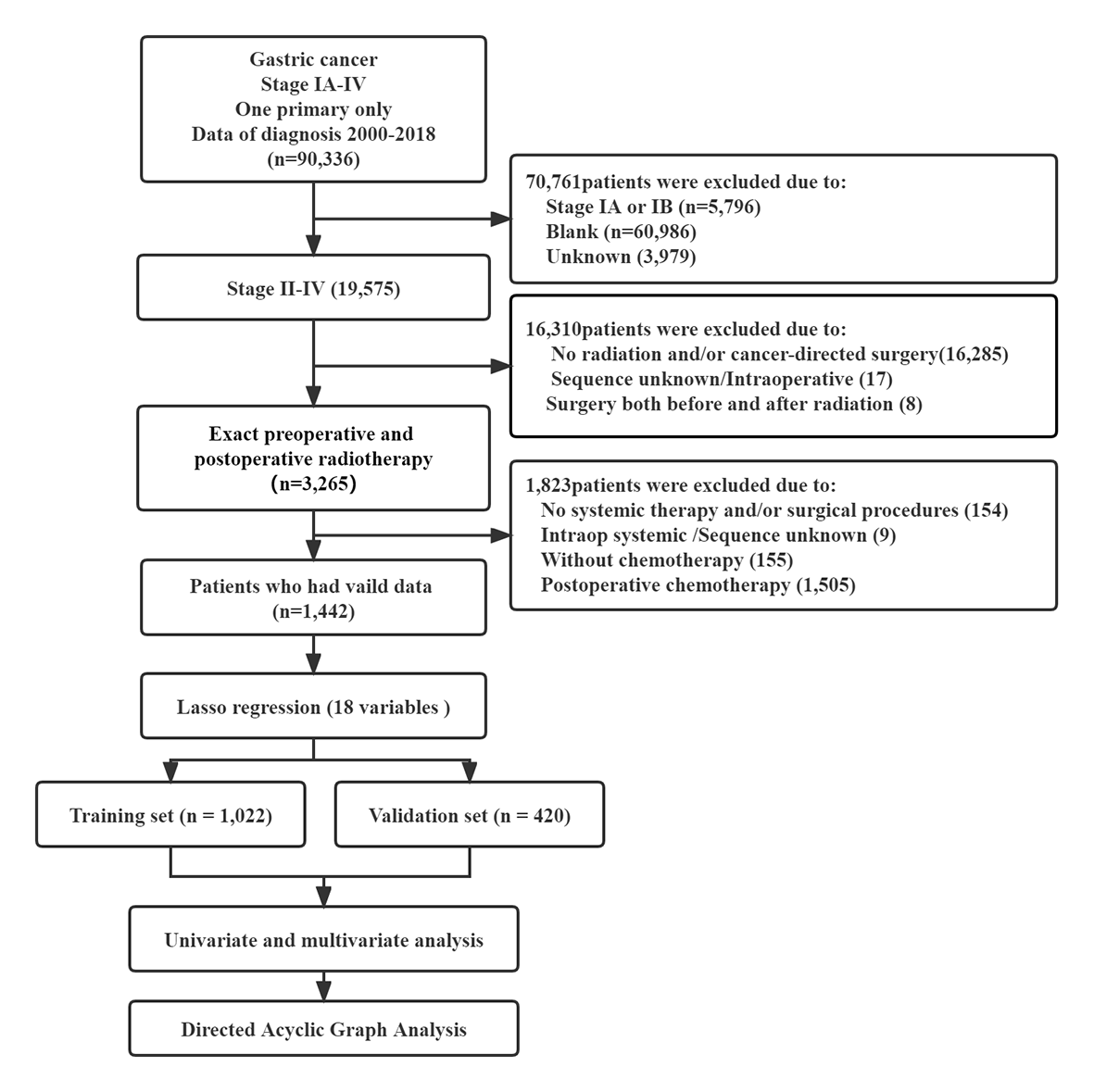

Supplement: S1 Fig — (TIF) [file pone.0283854.s001.tif]

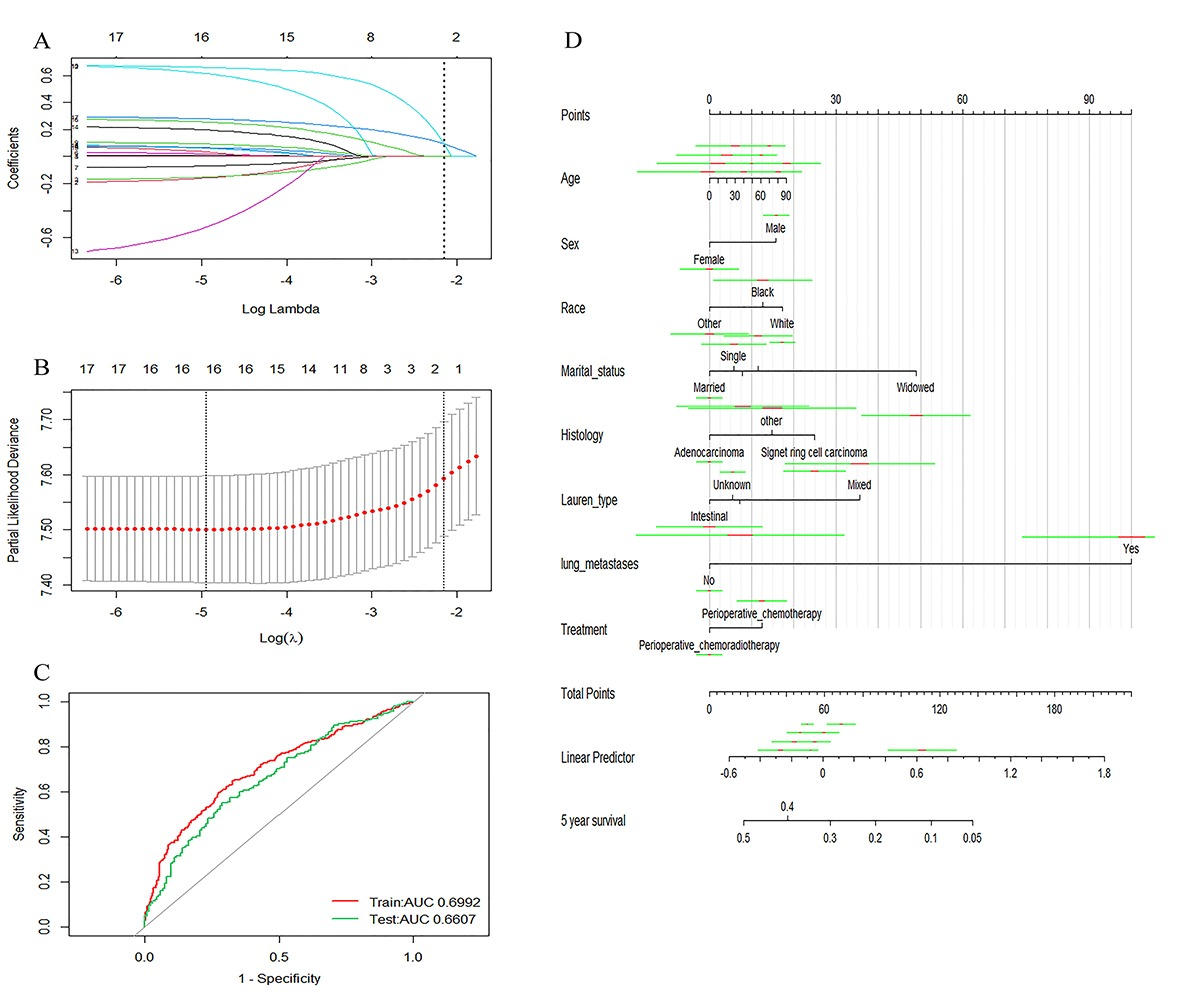

Supplement: S2 Fig — (A) Selection of tuning parameter (λ) in the LASSO regression using 10-fold cross-validation via minimum criteria. The partial likelihood binomial deviance is plotted vs log (λ). At the log (λ) of the optimal value, where features are selected, dotted vertical lines are set using the minimum criteria and the one standard error of the minimum criteria. (B) LASSO coefficient profiles for clinical features, each coefficient profile plot is produced vs log (λ) sequence. The dotted vertical line is set at the nonzero coefficients selected via 10-fold cross-validation, where sixteen nonzero coefficients are included. (C) The ROC curve is used to describe the statistical performance of the training set and test set model. (D) A nomogram for presenting 5-year probabilities of gastric cancer patients with the advanced stage was established. (TIF) [file pone.0283854.s002.tif]
