# Supplementary material for: Survival benefits of perioperative chemoradiotherapy versus chemotherapy for advanced stage gastric cancer based on directed acyclic graphs
Source: PLoS One. 2023 Apr 14;18(4):e0283854. doi: 10.1371/journal.pone.0283854 (PMC10104374; doi:10.1371/journal.pone.0283854)
Supplement: S1 Table — (DOCX) [file pone.0283854.s003.docx]

S1 Table. Basic Information and Clinicopathological Characteristics of Gastric Cancer Patients between the training set and the validation set

| Characteristic | Total (n=1442) | No. of Patients, n (%, row) | |  |
| --- | --- | --- | --- | --- |
|  |  | Training set  n=1022,(70.8%) | Validation set  n = 420,(29.2%) | *P* |
| Age (years) |  |  |  | 0.812 |
| ≤ 65 | 878 (60.9) | 620 (60.7) | 258 (61.4) |  |
| > 65 | 564 (39.1) | 402 (39.3) | 162 (38.6) |  |
| Sex |  |  |  | 0.347 |
| Male | 1153 (80.0) | 824 (80.6) | 329 (78.3) |  |
| Female | 289 (20.0) | 198 (19.4) | 91 (21.7) |  |
| Race |  |  |  | 0.320 |
| White | 1197 (83.0) | 857 (83.8) | 340 (81.0) |  |
| Black | 93 (6.5) | 65 (6.4) | 28 (6.6) |  |
| Other | 152 (10.5) | 100 (9.8) | 52 (12.4) |  |
| Marital status |  |  |  | 0.540 |
| Married | 974 (67.6) | 682 (66.7) | 292 (69.5) |  |
| Divorced/Separated | 157 (10.9) | 110 (10.8) | 47 (11.2) |  |
| Single | 202 (14.0) | 145 (14.2) | 57 (13.6) |  |
| Widowed | 64 (4.4) | 50 (4.9) | 14 (3.3) |  |
| Unknown | 45 (3.1) | 35 (3.4) | 10 (2.4) |  |
| Primary site |  |  |  | 0.532 |
| Cardiac/fundus | 1174 (81.4) | 836 (81.8) | 338 (80.5) |  |
| Body | 34 (2.4) | 22 (2.2) | 12 (2.8) |  |
| Antrum/pylorus | 79 (5.5) | 54 (5.3) | 25 (6.0) |  |
| Lesser/greater curvature | 75 (5.2) | 49 (4.7) | 26 (6.2) |  |
| Other | 80 (5.5) | 61 (6.0) | 19 (4.5) |  |
| Histology |  |  |  | 0.475 |
| Adenocarcinoma | 1157 (80.2) | 812 (79.5) | 345 (82.2) |  |
| Signet ring cell carcinoma | 206 (14.3) | 153 (15.0) | 53 (12.6) |  |
| Other | 79 (5.5) | 57 (5.5) | 22 (5.2) |  |
| TNM Stage |  |  |  | 0.392 |
| II | 441 (30.6) | 323 (31.6) | 118 (28.1) |  |
| III | 907 (62.9) | 632 (61.8) | 275 (65.5) |  |
| IV | 94 (6.5) | 67 (6.6) | 27 (6.4) |  |
| T stage |  |  |  | 0.020 |
| T1-2 | 209 (14.5) | 147 (14.3) | 62 (14.8) |  |
| T3 | 1053 (73.0) | 763 (74.7) | 290 (69.0) |  |
| T4 | 180 (12.5) | 112 (11.0) | 68 (16.2) |  |
| N stage |  |  |  | 0.238 |
| N0 | 319 (22.1) | 237 (23.2) | 82 (19.5) |  |
| N1 | 644 (44.7) | 457 (44.7) | 187 (44.5) |  |
| N2 | 329 (22.8) | 230 (22.5) | 99 (23.6) |  |
| N3 | 150 (10.4) | 98 (9.6) | 52 (12.4) |  |
| M stage |  |  |  | 1.000 |
| M0 | 1348 (93.5) | 955 (93.4) | 393 (93.6) |  |
| M1 | 94 (6.5) | 67 (6.6) | 27 (6.4) |  |
| Differentiation |  |  |  | 0.053 |
| Poorly | 822 (57.0) | 580 (56.8) | 242 (57.6) |  |
| Moderately | 417 (28.9) | 304 (29.7) | 113 (26.9) |  |
| Well | 45 (3.1) | 24 (2.3) | 21 (5.0) |  |
| Undifferentiated | 158 (11.0) | 114 (11.2) | 44 (10.5) |  |
| Summary stage |  |  |  | 0.514 |
| Regional | 1106 (76.7) | 776 (75.9) | 330 (78.6) |  |
| Distant | 222 (15.4) | 161 (15.8) | 61 (14.5) |  |
| Localized | 114 (7.9) | 85 (8.3) | 29 (6.9) |  |
| Lauren type |  |  |  | 0.925 |
| Intestinal | 75 (5.2) | 52 (5.2) | 23 (5.5) |  |
| Diffuse | 52 (3.6) | 38 (3.7) | 14 (3.3) |  |
| Mixed | 32 (2.2) | 24 (2.3) | 8 (1.9) |  |
| Unknown | 1283 (89.0) | 908 (88.8) | 375 (89.3) |  |
| Tumor size (cm) |  |  |  | 0.041 |
| ≤ 5 | 777 (53.9) | 529 (51.8) | 248 (59.0) |  |
| > 5 | 385 (26.7) | 285 (27.9) | 100 (23.8) |  |
| Unknown | 280 (19.4) | 208 (20.3) | 72 (17.2) |  |
| Bone metastases |  |  |  | 1.000 |
| Yes | 1381 (95.8) | 43 (4.2) | 18 (4.3) |  |
| No/Unknown | 61 (4.2) | 979 (95.8) | 402 (95.7) |  |
| Brain metastases |  |  |  | 0.872 |
| Yes | 3 (0.2) | 2 (0.2) | 1 (0.2) |  |
| No/Unknown | 1439 (99.8) | 1020 (99.8) | 419 (99.8) |  |
| liver metastases |  |  |  | 0.527 |
| Yes | 26 (1.8) | 20 (2.0) | 6 (1.4) |  |
| No/Unknown | 1416 (98.2) | 1002 (98.0) | 414 (98.6) |  |
| lung metastases |  |  |  | 0.525 |
| Yes | 11 (0.8) | 9 (0.9) | 2 (0.5) |  |
| No/Unknown | 1431 (99.2) | 1013 (99.1) | 418 (99.5) |  |
| Treatment |  |  |  | 0.645 |
| PCT | 410 (28.4) | 287 (28.1) | 123 (29.3) |  |
| PCRT | 1032 (71.6) | 735 (71.9) | 297 (70.7) |  |

Abbreviations: PCT, Perioperative chemotherapy; PCRT, Perioperative chemoradiotherapy.
