# Supplementary material for: Survival benefits of perioperative chemoradiotherapy versus chemotherapy for advanced stage gastric cancer based on directed acyclic graphs
Source: PLoS One. 2023 Apr 14;18(4):e0283854. doi: 10.1371/journal.pone.0283854 (PMC10104374; doi:10.1371/journal.pone.0283854)
Supplement: Attachment — Submitted filename: Comment-.docx [file pone.0283854.s005.docx]

Comment

It was pleasure to review the manuscript entitle,“Survival benefits of perioperative chemoradiotherapy based on directed acyclic graphs”. Though the manuscript is of scientific importance and can be useful, I have two major concerns: 1.the conclusion you have been made “PCRT may be feasible for these patients, however the analysis of literature with different results and conclusion from previous studies is lacking in discussion. 2. The focus of this paper, directed by the title and key words, includes the statistical analysis method directed acyclic graphs, which identifies the temporal causal structure of variables by analyzing the correlation and partial correlation coefficients between variables. Nodes and directed edges are then used to indicate the presence and directivity of causality. However, DAG accounts for a small proportion in the results, and the analysis of results do not account for its due proportion. Therefore, it is suggested to focus on innovative of DAG and its highlight results in discussion part. At the same time, the arrows in DAG represent causality. In the correlation figure of DAG, we can see that the outcome and the arrows of type are mutually correlated. Lauren type can affect the prognosis of patients, but the prognosis is farfetched as an influence factor of Lauren type. This part of the data content needs to be further clarified. Manuscript may be accepted once the question is corrected or included in the manuscript.
