# Supplementary material for: Survival benefits of perioperative chemoradiotherapy versus chemotherapy for advanced stage gastric cancer based on directed acyclic graphs
Source: PLoS One. 2023 Apr 14;18(4):e0283854. doi: 10.1371/journal.pone.0283854 (PMC10104374; doi:10.1371/journal.pone.0283854)
Supplement: Attachment — Submitted filename: Response to reviewers.doc [file pone.0283854.s006.doc]

**Dear** **Editors** **and reviewers**

# Thank you for your letter dated January 16. We were pleased to know that our work was rated as potentially acceptable for publication in Journal, subject to adequate revision. We thank the reviewers for the time and effort that they have put into reviewing the previous version of the manuscript. Their suggestions have enabled us to improve our work. Based on the instructions provided in your letter, we uploaded the file of the revised manuscript. Accordingly, we have uploaded a copy of the original manuscript with all the changes highlighted by using the track changes mode in MS Word. Appended to this letter is our point-by-point response to the comments raised by the reviewers. All changes made to the manuscript in response to the reviewer’s comments are highlighted in red. The page and line numbers mentioned in this response refer to those numbers in the revised manuscript.

We would like also to thank you for allowing us to resubmit a revised copy of the manuscript.

We hope that the revised manuscript is accepted for publication in the Journal of *PLOS ONE*

Sincerely,

Cheng Zheng

**Journal Requirements:**

1. Please ensure that your manuscript meets PLOS ONE's style requirements, including those for file naming.

**Response:** Thanks for your suggestion. We have made careful changes according to the format requirements of PLOS ONE.

2. (1) Please provide additional details regarding participant consent. In the ethics statement in the Methods and online submission information, please ensure that you have specified what type you obtained (for instance, written or verbal, and if verbal, how it was documented and witnessed). If your study included minors, state whether you obtained consent from parents or guardians. If the need for consent was waived by the ethics committee, please include this information.

(2) Your ethics statement should only appear in the Methods section of your manuscript. If your ethics statement is written in any section besides the Methods, please move it to the Methods section and delete it from any other section.

**Response:** Thank you for the suggestion. This section was revised and modified according to the information shown in the work suggested by the reviewer. Please see **Lines 106-109 on page 4** of the revised manuscript: The study used de-identified data and adhered to the World Medical Association's Declaration of Helsinki for Ethical Human Research. Informed consent was obtained from all subjects and their legal guardian.

3. (1) Thank you for stating in your Funding Statement: "This work was supported by Ningxia Natural Science Foundation. Item number: 2022AAC03173." Please provide an amended statement that declares *all* the funding or sources of support (whether external or internal to your organization) received during this study, as detailed online in our guide for authors at http://journals.plos.org/plosone/s/submit-now. Please also include the statement “There was no additional external funding received for this study.” in your updated Funding Statement. Please include your amended Funding Statement within your cover letter. We will change the online submission form on your behalf.

(2) Please state what role the funders took in the study.

**Response:** Thank you for the suggestion. We have removed the Funding Statement from the manuscript and added it to the cover letter.

4. Please complete your Competing Interests on the online submission form to state any Competing Interests.

**Response:** Thank you for the suggestion. We have removed the Competing Interests from the manuscript and added them to the cover letter.

5. Please upload a new copy of Figure 1 as the detail is not clear.

**Response:** Thank you for the suggestion. Figure 1 was modified according to the comment.

***REVIEWER 1 EVALUATION:***

1. The authors of the manuscript "survival benefits of perioperative chemoradiotherapy for the advanced stage of gastric cancer based on directed acyclic graphs" compared perioperative chemotherapy (PCT) and perioperative chemoradiotherapy (PCRT) on outcomes in patients with locally advanced gastric cancer and investigated the usefulness of directed acyclic graphs in this setting. There is a need for thorough language editing. I feel that I did not fully understand the methods of this work.

**Response:**

We apologize for the poor language of our manuscript. We worked on the manuscript for a long time and the repeated addition and removal of sentences and sections obviously led to poor readability. We have now worked on both language and readability and have sought help from AJE's English editing services to revise the whole manuscript to achieve standard publishing quality. We really hope that the flow and language level have been substantially improved. In addition, we have carefully proofread the manuscript to minimize typos and graphical, grammatical, and bibliographical errors in the Methods and added a description and explanation of the DAGs methods to make it easier to understand the article.

***REVIEWER 2 EVALUATION:***

1. On page 8, in line 176, are 410 patients without PCT?

**Response:** Thanks for your suggestion. We were sorry for our careless mistakes. We have addressed the above point in the sentence. Please see **Lines 173-175 on page 6** of the manuscript: A total of 1422 advanced GC patients including 289 females and 1153 males in the current study were composed of 410 patients without PCT and 1032 patients with PCRT.

2. In line 183, what CG means?

**Response:** This sentence was rephrased according to the comment. Please see **Lines 177-179 on page 6** of the manuscript: There were 822 patients with poor differentiation, accounting for 57.0% of all patients, and 1233 patients with an advanced T stage (T3-T4), accounting for 85.5% of all patients.

***REVIEWER 3 EVALUATION:***

1. Authors need to decide whether this paper’s objective is to approve DAG as a useful tool for comparing such outcomes or to find out the effect of PCRT. This point has to be clear in the introduction and after that, the whole manuscript storyline would change. If it is the first one, no changes are needed. But if it is the second one, please revise your introduction and conclusion.

**Response:** Thanks for your suggestion. The paper’s objective is to approve DAG as a useful tool for comparing such outcomes instead of finding out the effect of PCRT. We have reduced the proportion of discussions about the role of chemotherapy and chemoradiotherapy in advanced gastric cancer and increased the DAG-related content.

2. Introduction, line 79: I suggest starting with defining DAGs as useful tools rather than mentioning that it has been used in the current study. Also, it would be better if you could move this part completely to the methods section. Most of the audience of this paper would be clinicians that may not be interested in the statistical methodology. They might want to know the final outcome regardless of your statistical method.

(1) I suggest starting with defining DAGs as useful tools rather than mentioning that it has been used in the current study.

**Response:** Thanks for your suggestion. This sentence was rephrased according to the comment. Please see **Lines 84-85 on page 3** of the manuscript: Directed acyclic graphs (DAGs) are visual representations of causality in scientific discussions and are being increasingly used in modern epidemiology.

(2) We have removed the part of the DAGs-related content in the introduction and increased the proportion of advantages and applications about the DAGs-related content in the discussion.

3. Methods, lines 140 to 152: it is very uncommon to have a review of the literature within the methods section. If the authors are seeking to come up with some rationales behind their implemented method, they should do it in the discussion. Besides, references are added in the wrong fashion. Finally, the last sentence does not indicate at all what is your solution for the abovementioned confounding factors. Please revise this part.

**Response:** (1) We are grateful for the suggestion. We refer to serves typical articles on directed acyclic graphs in clinical practice and therefore put a review of the literature within the methods section^1-3^. To be more clear and in accordance with the reviewer's suggestion, we have added a brief discussion. (2) Thank you for underlining this deficiency, we have addressed the above point by the format of the references. (3) Confounders were not further explained because the purpose of this section is only to determine the bound and destination of the covariates derived from literature and expert knowledge. Confounders are presented in the results of our paper and are discussed in the discussion.

4. Discussion: I suggest adding some justifications and assumptions based on the current literature for the study’s findings. For example, why do you think male, white, and married individuals with tumor sizes of more than 5cm benefit more from PCRT?

**Response:** Thank you for underlining this deficiency. This section was revised and modified according to the information shown in the work suggested by the reviewer. Please see **Lines 263-286 on page 13** of the discussion section: Findings from our study indicate that PCRT benefits patients who are age ≤ 65, male sex, white race, or have regional tumors. An analysis of the SEER database from 2005 to 2016 confirms earlier findings that age is a poor prognostic factor and that PCT and age greater than 60 years are related to a worse outcome. Patients who were 60 years or older had a 5-year OS that was nearly 30% lower than those who were younger. In addition, there is an ethnic disparity in PCT use and outcomes among GC patients in the United States. After controlling for patient/disease/hospital factors, race was independently associated with less PCT use4. There was no difference in OS between patients of different ethnicities. Another study demonstrated that GC patients' comorbidity profiles varied racially and ethnically from those of the matched cancer-free group. In particular, Whites and Blacks had higher rates of comorbid conditions than Asians or Pacific Islanders5. The study also discovered that anemia was the most prevalent organ, blood, and metabolic condition among non-White GC patients who were treated with CT within 6 months after diagnosis. The incidence rates were more than twice as high among Chinese, Japanese, and Filipinos as they were among Whites, Blacks, and Hispanics. The optimal therapy for locally advanced GC is perioperative multidisciplinary treatment, including PCT, RT, immunotherapy, and targeted therapy; therefore, comprehensive treatment is paramount to treatment selection6. In the West, PCT and PCRT are recommended for resectable patients with regional tumor invasion into the nearby tissues or middle lymph node metastasis, since their SWOG/INT-0116 trial demonstrated that the OS and recurrence-free survival (RFS) of GC patients who have had R0 resection followed by PCRT (45 Gy of RT combined with bolus fluorouracil [FU] and leucovorin) were longer than the OS and RFS of those who had surgery alone (5-year OS, 40% vs. 30%, respectively; 5-year RFS, 48% vs. 31%, respectively)7. These differences, such as age, race, and the presence of a regional tumor, may have an impact on the curative effect of PCRT.

***REVIEWER 4 EVALUATION:***

1. Manuscript Type: Retrospective Study In this study, the authors explore the Pros and cons between PCT and PCRT, constructed multivariate analysis DAG model to analyze the factors affecting the prognosis of patients. As for language and narrative skills, the overall reading experience is smooth.

**Response:** We thank the reviewer for the time and effort that they have put into reviewing the previous version of the manuscript. To revise the whole manuscript to achieve standard publishing quality. We have now worked on both language and readability and have sought help from AJE's English editing services to revise the whole manuscript. In addition, we point-by-point responses to the comments raised by the reviewers and throughout a substantial adjustment, so that they can be successfully published. We would like to express our heartfelt thanks to the reviewers for their affirmation of the article.

***REVIEWER 5 EVALUATION:***

2. First, many major grammatical mistakes made a considerable part of the manuscript ambiguous, and the English language of the manuscript needs major revisions.

**Response:** We apologize for the poor language of our manuscript. We worked on the manuscript for a long time and the repeated addition and removal of sentences and sections obviously led to poor readability. We have now worked on both language and readability and have sought help from AJE's English editing services to revise the whole manuscript to achieve standard publishing quality. We really hope that the flow and language level have been substantially improved.

3. Second, the use of causal inference methods, such as DAG, can face some limitations. The feature selection method will minimize the overfitting issue of DAG to some extent, but Although the authors sufficiently mentioned limitations of DAG in their “limitations”, the conclusions may mislead the audience.

**Response:** We are grateful for the suggestion. To be more clear and follow the reviewer's concerns and the results were revised and modified according to the information shown in the work suggested by the reviewer. Please see **Lines 231-239 on page 11** of the revised manuscript: Five variables directly influenced the primary survival outcome: sex (r=-0.0676, HR=1.032), marital status (r=0.0268, HR=1.021), histology type (r=0.0565, HR=1.015), lung metastases (r=0.3200, HR=1.024), and treatment (r=-0.0992, HR=1.001). Four of these variables directly influenced the secondary outcome measure treatment: sex (r=-0.0676, HR=1.001), race (r=-0.1314, HR=1.001), histology type (r=-0.1032, HR=1.001) and Lauren type (r=-0.0318, HR=1.040). Sex, race, and Lauren type were associated with the primary survival outcome, but these factors were not associated with the secondary outcome measure treatment (Table 2, all P<0.05), which indicated that these may be confounding factors by comparing the results with the Cox regression analysis.

4. Third, some points are missed in the method section, and the representation of DAG (Figure 3) is not informative enough. I made several comments below which might be helpful for the authors to improve their manuscript.

(1) the conclusion you have made is “PCRT may be feasible for these patients, however, the analysis of literature with different results and conclusions from previous studies is lacking in the discussion.

**Response:** Thank you for underlining this deficiency. This section was revised and modified according to the information shown in the work suggested by the reviewer (Line 41, page 3). Please see **Lines 263-285 on page 13** of the discussion section: Findings from our study indicate that PCRT benefits patients who are age ≤ 65, male sex, white race, or have regional tumors. An analysis of the SEER database from 2005 to 2016 confirms earlier findings that age is a poor prognostic factor and that PCT and age greater than 60 years are related to a worse outcome. Patients who were 60 years or older had a 5-year OS that was nearly 30% lower than those who were younger. In addition, there is an ethnic disparity in PCT use and outcomes among GC patients in the United States. After controlling for patient/disease/hospital factors, race was independently associated with less PCT use4. There was no difference in OS between patients of different ethnicities. Another study demonstrated that GC patients' comorbidity profiles varied racially and ethnically from those of the matched cancer-free group. In particular, Whites and Blacks had higher rates of comorbid conditions than Asians or Pacific Islanders5. The study also discovered that anemia was the most prevalent organ, blood, and metabolic condition among non-White GC patients who were treated with CT within 6 months after diagnosis. The incidence rates were more than twice as high among Chinese, Japanese, and Filipinos as they were among Whites, Blacks, and Hispanics. The optimal therapy for locally advanced GC is perioperative multidisciplinary treatment, including PCT, RT, immunotherapy, and targeted therapy; therefore, comprehensive treatment is paramount to treatment selection6. In the West, PCT and PCRT are recommended for resectable patients with regional tumor invasion into the nearby tissues or middle lymph node metastasis, since their SWOG/INT-0116 trial demonstrated that the OS and recurrence-free survival (RFS) of GC patients who have had R0 resection followed by PCRT (45 Gy of RT combined with bolus fluorouracil [FU] and leucovorin) were longer than the OS and RFS of those who had surgery alone (5-year OS, 40% vs. 30%, respectively; 5-year RFS, 48% vs. 31%, respectively)7. These differences, such as age, race, and the presence of a regional tumor, may have an impact on the curative effect of PCRT.

(2) DAG accounts for a small proportion of the results, and the analysis of results does not account for its due proportion.

**Response:** Thanks for your suggestion. We have increased the proportion of the DAG-related content in the results and discussion section. Please see **Lines 224-238 on page 11** of the result section: Based on our univariate and multivariate analysis, the following variables were included in the DAG analysis: sex, age, race, marital status, histologic type, Lauren type, lung metastases, and comprehensive treatment. Although DAGs based on literature or prior knowledge can intuitively reflect the outcomes and cause the path of each variable in the advanced GC, it does not mirror which pathway is significant. Therefore, it is indispensable to test the DAGs (Table 2). Five variables directly influenced the primary survival outcome, Sex (r=-0.0676, HR=1.032), Marital Status (r=0.0268, HR=1.021), Histology Type (r=0.0565, HR=1.015), Lung Metastases (r=0.3200, HR=1.024), and Treatment (r=-0.0992, HR=1.001). Four of these variables did directly influence the secondary outcome measure treatment, respectively, Sex (r=-0.0676, HR=1.001), Race (r=-0.1314, HR=1.001), Histology Type (r=-0.1032, HR=1.001) and Lauren Type (r=-0.0318, HR=1.040).

Please see **Lines 322-332 on pages 14-15** of the discussion section: DAGs, a visual representation of causal assumptions, are used by researchers to better examine confounding biases related to causal questions8, 9. DAGs may be preferable to the conventional definition of confounding, particularly in more complicated situations, as they enable the identification of the presumptive causal mechanism and, consequently, the possibility of collider-stratification bias with certain adjustments, as well as a minimal set of factors to adjust for to remove unwanted confounding10. Several tutorials and reviews on the use of DAGs have been published to aid technicians and clinicians11, 12. Lederer et al. reviewed the general concepts, including confounding and selection bias, for researchers in pulmonary medicine13. By doing so, a methodical technique to deliver a summary of the context and the causal research is presented. DAGs clarify the underlying relationships and act as a visual representation of causal assumptions14. DAGs can thus be used to clarify confusion and suggest solutions.

***1. GENERAL COMMENTS:***

1-5 The English language of the manuscript needs major revision.

**Response:** Thanks for your suggestion. We have carefully proofread the manuscript to minimize typos and graphical, grammatical, and bibliographical errors in the Methods and added a description and explanation of the DAGs methods to make it easier to understand the article. Moreover, we have sought help from AJE's English editing services to revise the whole manuscript to achieve standard publishing quality.

***2. In the TITLE and ABSTRACT section:***

2-1 The title indicated a summary of what was done with a commonly used term, however, it needs to be more specified. Since the survival benefits of perioperative chemoradiotherapy in comparison to perioperative chemotherapy were evaluated, it is suggested to change “survival benefits of perioperative chemoradiotherapy” to “survival benefits of perioperative chemoradiotherapy versus chemotherapy”.

**Response:** Thank you for the title suggested. The precedent version of the title has been replaced, Please see **Lines 2-4 on page 1**, becoming “Survival benefits of perioperative chemoradiotherapy versus chemotherapy for the advanced stage of gastric cancer based on directed acyclic graphs”.

2-2 In the abstract, the aim is not mentioned in detail and correctly. First, please change “GC patients” to “survival of patients with locally advanced GC”. Second, it seems better to change “construct directed acyclic graphs (DAGs) to evaluate the patient prognosis and disease risk” to “determine the factors affecting survival rate using directed acyclic graphs (DAGs)”.

**Response:** Thanks for your suggestion. We have modified the sentence according to the previous comment. Please see **Lines 23-24 on page 1** of the manuscript: The aim of this study was to compare the benefits of PCT and PCRT in GC patients and determine the factors affecting survival rate using directed acyclic graphs (DAGs).

2-3 Since the study just evaluated the survival rate and prognostic outcomes –not the risk of disease development-, the “disease risk of patients” located in line 32 should be removed.

**Response:** This phrase was modified according to the comment. Please see **Lines 29-31 on pages 1-2:** Third, corrective analyses for confounding factors were selected based on DAGs that show the possible association between advanced GC patients and outcomes and evaluate the prognosis.

2-3 In the “results” section, it is suggested to present survival rates in patients receiving PCRT or PCT, instead of “PCRT was significantly associated with the advanced GC compared to PCT for good prognosis”. Moreover, presenting a summary of baseline and clinical variables is suggested.

**Response:** Thanks for your suggestion. We have addressed the above point in the results section. Please see **Lines 31-34 on page 2** of the manuscript: Patients who received PECT had longer OS than those with POCT treatment (Hazard ratio = 0.846, 95% CI= 0.738-0.970, P = 0.015). The median overall survival of the PCRT group was 36.5 (15.0- 53.0) months higher than in, while that of the PCT group was 34.6 (16.0 - 48.0) months.

2-4 Since there are several grammatical mistakes, using English editing services to revise the whole manuscript to achieve standard publishing quality is strongly recommended.

**Response:** We apologize for the poor language of our manuscript. We worked on the manuscript for a long time and the repeated addition and removal of sentences and sections obviously led to poor readability. We have now worked on both language and readability and have sought help from AJE's English editing services to revise the whole manuscript to achieve standard publishing quality. We really hope that the flow and language level have been substantially improved.

***3. In the INTRODUCTION section:***

3-1 In line 50, the authors stated that “Due to the high heterogeneity of GC in histopathology and molecular biology, the survival prognosis is dismal” which seems to be incorrect and irrelevant. Although molecular and histopathological features of tumors have a significant role in determining the patient’s survival, however, the question is, are the variation and heterogenicity of these features responsible for the dismal prognosis of gastric cancer?

**Response:** Thanks for your suggestion, we have corrected the statements in the text accordingly. Please see **Lines 47-49 on page 2** of the manuscript: Because advanced-stage GC has largely heterogeneous characteristics, an uncertain mechanism, limited therapy strategies, and a less than 12-month median survival time[3-5], it is essential to develop optimal individualized management strategies for such patients.

3-2 In line 54, please change “advanced stage of patients” to “patients with advanced stage of GC”.

**Response:** Thanks for your suggestion. We have modified the sentence according to the previous comment. Please see **Lines 48-49 on page 2** of the manuscript: it is essential to develop optimal individualized management strategies for such patients.

3-3 In line 67, please describe in detail the “certain benefits”. Please discuss exactly what benefits were obtained.

**Response:** This phrase was modified according to the comment. Please see **Lines 52-53 on page 3** of the manuscript: leading to efforts to improve the survival of these patients receiving neoadjuvant or adjuvant therapies.

3-4 In line 73, please remove the word “direct” from “direct evidence”.

**Response:** Thanks for your suggestion, this phrase was modified according to the comment. Please see **Line 67 on page 3** of the manuscript.

3-5 In line 74, the authors said that “Until now, direct evidence comparing PCT and PCRT has been limited to several small randomized trials and was mainly based on East Asian countries rather than North Americans”. Please discuss a little about the results and limitations of these previous studies.

**Response:** Thanks for your suggestion, we have corrected the statements in the text accordingly. Please see **Lines 70-73 on page 3** of the manuscript: In Europe and the United States, it remains unclear whether PCT should be used in combination with radiotherapy and whether chemoradiotherapy is efficacious.

***4. In the METHODS section:***
4-1 In the “population” section, it is recommended to describe more details about the SEER database. Moreover, it is strongly recommended to determine the type/design of the present study.

**Response:** Thanks for your suggestion, we have corrected the statements in the text accordingly. Please see **Lines 97-100 on page 4** of the manuscript: The available data from a retrospective cohort study were extracted using SEER*stat (8.3.6) software. In our study, we used the SEER database's tumor nomenclature and coding manual[25] as well as the International Classification of Diseases tumor morphology code ICD-O-3 to extract data from 2004-2018 GC patients for therapeutic strategies[26]

4-2 It is recommended to estimate and present the mean/median follow-up duration.

**Response:** Thanks for your suggestion, we looked carefully at the original data and found no information on patient follow-up.

4-3 In the “Defining covariates for a directed acyclic graph” section, please describe the reasons you chose DAGs for evaluating factors that influence the GC prognosis since there are many other methods through which the effect of factors on disease prognosis and the outcome can be evaluated.

**Response:** Thanks for your suggestion, we have described the reasons why chose DAGs for evaluating factors that influence the GC prognosis in the introduction and discussion. Please see **Lines 73-82 on page 3** of the manuscript: Determining causal relations and eliminating bias are the primary aims of research conducted by the scientific community. To date, observational research has primarily focused on relationships between covariates and outcomes, with authors only rarely asserting a direct causal relationship. Because randomization has been regarded as the most reliable strategy for eliminating bias among treatment groups, statements of direct causal relationships have only been made in the context of randomized trials. Although randomized clinical trials are useful for addressing bias, they are expensive, time-consuming, frequently unfeasible, and unrepresentative of the intended audience when blinding methods are used[19, 20]. Given that observational research has enormous unrealized potential to guide clinical decision-making, structured approaches to examine causal connections in specified datasets are being increasingly noticed[21].

4-4 In line 100, the authors said that “All the data used in this study were derived from the SEER database with an openly accessible approach, so the release of data did not require a local ethics approval or a statement.”. It is important to note that although the retrospective design of the study, de-identifying patients, and using an openly accessible database may waive the need for gathering signed informed consent from the participants, it does not relieve the need for obtaining ethical approval.

**Response:** We have addressed the above point by the ethics statement in the Methods. Please see **Lines 107-109 on page 4** of the revised manuscript: The study used de-identified data and adhered to the World Medical Association's Declaration of Helsinki for Ethical Human Research. Informed consent was obtained from all subjects and their legal guardian.

4-5 In line 104, the authors said that patients with “stage II-IV” were selected. Since there are various guidelines and criteria for staging the cancers, it is necessary to mention that in the current study/database, what criteria were used for cancer staging and what are the clinical features of patients with stage II-IV GC (regarding tumor grading, invasion, metastasis, lymph node involvement, etc.).

**Response:** Thanks for your suggestion, we have added the statements in the text accordingly. Please see **Lines 101-102 on page 4** of the manuscript: In TNM staging was used to classify all cancer samples American Joint Committee on Cancer (AJCC).

4-6 In line 125, please change “OS” to “overall survival (OS)”. In addition, the whole manuscript states the abbreviations when they first appear in the text. Also, prevent mentioning the full abbreviation after your first use (Referring to Line 156).

**Response:** Thanks for your suggestion, we have corrected the statements in the text accordingly. Please see **Line 126 on page 5** and **Line 154 on page 6** of the manuscript

4-7 In line 141, the “The clinicopathological characteristics showed that younger patients with gastric adenocarcinoma have the more advanced and metastatic disease” seems wrong and needs correction and clarification.

**Response:** This phrase was modified according to the comment. Please see **Lines 177-179 on page 6** of the manuscript: There were 822 patients with poor differentiation, accounting for 57.0% of all patients, and 1233 patients with an advanced T stage (T3-T4), accounting for 85.5% of all patients.

5-8 In line 148, it is better to remove the “vary between races” and mention the effect of race on cancer prognosis in previous lines where demographic factors –not clinicopathological factors- are discussed –as well as the effect of the marriage status.

**Response:** Thanks for your suggestion, we have corrected the statements in the text accordingly. Please see **Lines 138-139 on page 5** of the manuscript:

5-9 In line 160, what does “the most significant variables” means statistically? Please use exact statistical definitions in the “statistical analysis” section.

**Response:** This phrase was modified according to the comment. Please see **Lines 156-158 on page 6** of the manuscript: Second, the variables of statistical significance were selected by the LASSO from the training set and then used in the univariate analysis.

5-10 In line 163, please change the “Independent” to “independent”, and use correct capitalization in your whole manuscript (In line 167 “Analyses” to “analysis”, …)

**Response:** Thanks for your suggestion, we have corrected the capitalization in the manuscript.

5-11 In line 169, please mention the statistical significance level of the p-value.

**Response:** Thanks for your suggestion, we have corrected the statements in the text accordingly. Please see **Lines 165-166 on page 6** of the manuscript: The p values were derived from two-tailed tests, and p values< 0.05 was considered statistically significant.

5-12 Authors used multivariate analysis, while they did not mention how they performed such analysis and how they included variables from univariate analysis.

**Response:** Thanks for your suggestion, we have corrected the statements in the text accordingly.

***5. In the RESULT section:***
In this section, several misreported measures need corrections.
5-1 In line 176, “A total of 1422 advanced GC patients including 289 females and 1153 males in the current study were composed of 410 patients without PCT and 1032 patients with PCRT” needs correction.

**Response:** Thanks for your suggestion. We were sorry for our careless mistakes. We have addressed the above point in the sentence. Please see **Lines 173-175 on page 6** of the manuscript: A total of 1422 advanced GC patients including 289 females and 1153 males in the current study were composed of 410 patients without PCT and 1032 patients with PCRT.

5-2 In line 179, the authors said that “In the majority of patients, 1174 patients (81.4%), the tumor was located at the antrum or pylorus”, while according to supplementary table 1, the majority of patients (n=1174) had tumors located in gastric cardiac/fundus area.

**Response:** Thanks for your suggestion, we have corrected the statements in the text accordingly. Please see **Lines 176-178 on page 6** of the manuscript: The mean age was 61±11 years. In most of the patients, 1174 patients (81.4%), the tumor was located at the cardiac or fundus, and regional tumors were reported in 1106 patients (76.7%).

5-3 In line 181, please change the “regional” to “regional tumors”.

**Response:** Thanks for your suggestion, we have corrected the statements in the text accordingly. Please see **Line 201 on page 7** of the manuscript.

5-4 In line 181, please change the “GC was most frequently poor differentiation” to “The majority of gastric tumors were poorly differentiated”.

**Response:** Thanks for your suggestion, this phrase was modified according to the comment.

5-5 In line 204, what does “visible heterogenicity” mean?

**Response:** Thanks for your suggestion, this sentence was rephrased according to the comment. Please see **Lines 191-192 on page 7:** The nomogram in this study only presents the independent risk factors in the multivariate analysis.

5-6 In line 215, “Notably, PCRT was still significantly associated with the advanced GC for good prognosis” needs correction. It is suggested to use this: “PCRT was still significantly associated with a better survival in patients with advanced GC”.

**Response:** Thanks for your suggestion, we have corrected the statements in the text accordingly. Please see **Lines 213-215 on page 11** of the manuscript: PCRT was still significantly associated with better survival in patients with advanced GC (HR = 0.862, 95% CI= 0.744-0.996, P =0.044).

5-7 many parts are ambiguous and meaningless:
In line 219, the “show the possible relationship with advanced GC patients” is wrong and meaningless and needs correction.

**Response:** Thanks for your suggestion, we have corrected the statements in the text accordingly.

In line 221, “cause the path of each variable in the advanced GC, it does not mirror which pathway is significant” is wrong and meaningless and needs correction.

**Response:** Thanks for your suggestion, we have corrected the statements in the text accordingly. Please see **Lines 228-230 on page 11** of the manuscript: Although DAGs based on literature or prior knowledge can intuitively reflect the status and path of each variable in the advanced GC, it is not clear which pathway is meaningful to intuitively reflect, and therefore it is indispensable to test the DAGs (Table 2).

In line 222, “The prognosis of sex, marital status, histology type, lung metastases, and treatment were significantly associated with advanced GC in both cox regression models and DAG tests” is wrong and meaningless and needs correction.

**Response:** Thanks for your suggestion, we have removed this sentence in the text.

In line 225, the “Additionally, DAGs results showed that the factors of sex, race, histology type, and Lauren type impact the effect to administer treatment” is wrong and meaningless and needs correction.

**Response:** Thanks for your suggestion, we have corrected the statements in the text accordingly. Please see **Lines 235-238 on page 11** of the manuscript. Sex, race, and Lauren type were associated with the primary survival outcome, but these factors were not associated with the secondary outcome measure treatment (Table 2, all P<0.05), which indicated that these may be confounding factors by comparing the results with the Cox regression analysis.

***6. In the DISCUSSION and CONCLUSION section:***

6-1 The discussions should be rewritten since it is monotonous and not informative.
**Response:** We are grateful for the suggestion. To be more clear and in accordance with the reviewer's concerns, we have rewritten the discussion section according to the reviewer's comments. Please see **Lines 249-332 on pages 12-15** of the manuscript.

“In this cohort study, the SEER database was used to retrospectively analyze the demographic and clinical characteristics of patients with locally advanced GC to evaluate the effect of PCT and PCRT on the prognosis of such patients. The results indicated that PCRT had significant survival benefits when compared to PCT for patients with advanced GC. In a study using the SEER database, 21,447 patients with stage I-IV GC benefited most from adjuvant RT with CT when compared with surgery or PCT[49]. In the multivariate Cox regression model, male sex, widowed status, signet ring cell carcinoma, and lung metastases were considered to be independent risk factors for a poor prognosis. Sex, race, and Lauren type were associated with the primary survival outcome, but these factors were not associated with the secondary outcome measure treatment by DAG methods, which indicated that these may be confounding factors by comparing the results with the Cox regression analysis.

The prognosis of CRT in GC patients has been reported in several previous randomized studies. The INT0116 study suggested that adjuvant CT combined with RT was effective for individuals with specific treatment modalities and disease pathological stages [12]. Findings from our study indicate that PCRT benefits patients who are age ≤ 65, male sex, white race, or have regional tumors. An analysis of the SEER database from 2005 to 2016 confirms earlier findings that age is a poor prognostic factor and that PCT and age greater than 60 years are related to a worse outcome. Patients who were 60 years or older had a 5-year OS that was nearly 30% lower than those who were younger. In addition, there is an ethnic disparity in PCT use and outcomes among GC patients in the United States. After controlling for patient/disease/hospital factors, race was independently associated with less PCT use[11]. There was no difference in OS between patients of different ethnicities. Another study demonstrated that GC patients' comorbidity profiles varied racially and ethnically from those of the matched cancer-free group. In particular, Whites and Blacks had higher rates of comorbid conditions than Asians or Pacific Islanders[56]. The study also discovered that anemia was the most prevalent organ, blood, and metabolic condition among non-White GC patients who were treated with CT within 6 months after diagnosis. The incidence rates were more than twice as high among Chinese, Japanese, and Filipinos as they were among Whites, Blacks, and Hispanics. The optimal therapy for locally advanced GC is perioperative multidisciplinary treatment, including PCT, RT, immunotherapy, and targeted therapy; therefore, comprehensive treatment is paramount to treatment selection[42]. In the West, PCT and PCRT are recommended for resectable patients with regional tumor invasion into the nearby tissues or middle lymph node metastasis, since their SWOG/INT-0116 trial demonstrated that the OS and recurrence-free survival (RFS) of GC patients who have had R0 resection followed by PCRT (45 Gy of RT combined with bolus fluorouracil [FU] and leucovorin) were longer than the OS and RFS of those who had surgery alone (5-year OS, 40% vs. 30%, respectively; 5-year RFS, 48% vs. 31%, respectively)[57]. These differences, such as age, race, and the presence of a regional tumor, may have an impact on the curative effect of PCRT.

The improved survival observed with PCT could be attributed to tumor regression. Multiple trials have shown that PCRT reduces the primary tumor volume and the local tumor recurrence rate and increases the rates of pathological complete response, negative margin resection, and overall survival[8, 42]. A study from South Korea showed that progression-free survival (PFS) in patients who received neoadjuvant therapy (TNT) was significantly improved when compared with that in patients who received neoadjuvant chemoradiotherapy (NCRT), and the OS tended to be longer without any side effects[58]. The Neo-PLANET study analyzed the safety and efficacy of ocrelizumab combined with CRT in the neoadjuvant treatment of locally advanced proximal gastric adenocarcinoma. The interim analysis showed a 91.7% R0 resection rate, 12 patients achieved pathological complete response (PCR, 33.3%), and the rate of major pathological response (MPR) was 41.7%[59]. Additionally, there is a potential benefit to treating micrometastatic disease and decreasing tumor cell spread following resection[8]. However, subsets of individuals might not benefit more from CRT owing to poor tolerance and limited toxicity. A study showed that up to 50% of patients who underwent surgical resection never received CRT, presumably owing to poor tolerance, whereas neoadjuvant therapy may be better tolerated and more reliably applicable[52]. In recent years, technical advances have enabled oncologists to deliver precise doses of RT and minimize exposure to critical organs, resulting in improved therapeutic effects and decreased toxicity.

For reliable causal inferences to be drawn from observational data, confounding needs to be appropriately addressed because it hides the true effect of the exposure. DAG analysis shows that age, race, and Lauren type are integral to the impact of comprehensive treatment, meaning that these factors indirectly affect the prognosis of patients with advanced-stage disease because of the direct impact on the treatment modality, so they could cause confounder bias in regression analyses. GC is considered an age-related disease, with the majority of newly diagnosed patients in the United States being over 75 years old. Elderly patients frequently have restricted inclusion in clinical trials owing to the physiological changes that occur with age, including pharmacodynamic variability, diminished organ function, and impaired functional status, which necessitate individualized treatment approaches. A study reported survival advantages in patients younger than 70 years old who received adjuvant CRT but not in those older than 70 years old [37]. Additionally, a study noted that CRT improved the outcomes of patients with the intestinal type when compared with those with the diffuse type. Although the reason for the difficulties in locoregional control of diffuse GC is yet to be discovered, the benefit of local control may be associated with a better survival rate. These differences call for the exploration of multiple approaches in the potentially curative treatment of advanced GC. Therefore, it is possible to consider that age, race, and Lauren type may be confounding factors affecting prognosis in the advanced stage.

DAGs, a visual representation of causal assumptions, are used by researchers to better examine confounding biases related to causal questions[26, 27]. DAGs may be preferable to the conventional definition of confounding, particularly in more complicated situations, as they enable the identification of the presumptive causal mechanism and, consequently, the possibility of collider-stratification bias with certain adjustments, as well as a minimal set of factors to adjust for to remove unwanted confounding[60]. Several tutorials and reviews on the use of DAGs have been published to aid technicians and clinicians[28, 29]. Lederer et al. reviewed the general concepts, including confounding and selection bias, for researchers in pulmonary medicine[30]. By doing so, a methodical technique to deliver a summary of the context and the causal research is presented. DAGs clarify the underlying relationships and act as a visual representation of causal assumptions[61]. DAGs can thus be used to clarify confusion and suggest solutions”.

In line 267, the authors said that PCRT was associated with “local tumor recurrence” in previous studies. Since local tumor recurrence is considered an adverse outcome, it seems that this sentence is wrong and needs correction. Please again check the findings of the mentioned studies and revise this sentence.

**Response:** Thanks for your suggestion, we have corrected the statements in the text accordingly. Please see **Lines 286-289 on page 13** of the manuscript. Multiple trials have shown that PCRT reduces primary tumor volume and local tumor recurrence rate, and increases rates of pathological complete response, negative margin resection and overall survival.

***7. Tables and Figures:***
The tables do not have appropriate titles.

7-1 In Table 1, there are many mistakes in presenting the numbers/sizes of each group/variable. Are the numbers in the parenthesis showing the percentage?

**Response:** Thanks for your suggestion, this table was modified according to the comment. Please see **Line 203 on pages 8-10** of the manuscript. The numbers in the parenthesis show the Mortality.

7-2 In Table 1, the sum of participants in subgroups does not match the total participant in many cases. Please add a row for missing values, if it is the case.

**Response:** Thanks for your suggestion, the table was modified according to the comment.

7-3 In Figure 3, the structure of the variables is ambiguous.

**Response:** Thanks for your suggestion, the figure was modified according to the comment.

***8. References:***

8-1 A majority of references are old (do not belong to the recent 10 years). It is strongly suggested to use references that are published in recent 10 years if possible.

**Response:** We are grateful for the suggestion. We have carefully updated the References as much as possible within 8 years.

**References**

1.	Röhrig N, Strobl R, Müller M, Perz S, Kääb S, Martens E, et al. Directed acyclic graphs helped to identify confounding in the association of disability and electrocardiographic findings: results from the KORA-Age study. J Clin Epidemiol. 2014;67(2):199-206.

2.	Hoorntje A, Kuijer P, van Ginneken BT, Koenraadt KLM, van Geenen RCI, Kerkhoffs G, et al. Prognostic Factors for Return to Sport After High Tibial Osteotomy: A Directed Acyclic Graph Approach. The American journal of sports medicine. 2019;47(8):1854-62.

3.	Etminan M, Collins GS, Mansournia MA. Using Causal Diagrams to Improve the Design and Interpretation of Medical Research. Chest. 2020;158(1s):S21-s8.

4.	Ikoma N, Cormier JN, Feig B, Du XL, Yamal JM, Hofstetter W, et al. Racial disparities in preoperative chemotherapy use in gastric cancer patients in the United States: Analysis of the National Cancer Data Base, 2006-2014. Cancer. 2018;124(5):998-1007.

5.	Lowe KA, Danese MD, Gleeson ML, Langeberg WJ, Ke J, Kelsh MA. Racial and Ethnic Variability in the Prevalence and Incidence of Comorbidities Associated with Gastric Cancer in the United States. Journal of gastrointestinal cancer. 2016;47(2):168-81.

6.	Joshi SS, Badgwell BD. Current treatment and recent progress in gastric cancer. CA Cancer J Clin. 2021;71(3):264-79.

7.	Ma GF, Zhang HG, Liu J, Chen YX, Xiao H, Wang XF, et al. Benefit of adjuvant chemoradiotherapy in patients with pathological stage III gastric cancer. Cancer management and research. 2019;11:6029-41.

8.	Mansournia MA, Hernán MA, Greenland S. Matched designs and causal diagrams. International journal of epidemiology. 2013;42(3):860-9.

9.	Mansournia MA, Higgins JP, Sterne JA, Hernán MA. Biases in Randomized Trials: A Conversation Between Trialists and Epidemiologists. Epidemiology (Cambridge, Mass). 2017;28(1):54-9.

10.	Suttorp MM, Siegerink B, Jager KJ, Zoccali C, Dekker FW. Graphical presentation of confounding in directed acyclic graphs. Nephrology, dialysis, transplantation : official publication of the European Dialysis and Transplant Association - European Renal Association. 2015;30(9):1418-23.

11.	Stovitz SD, Shrier I. Causal inference for clinicians. BMJ evidence-based medicine. 2019;24(3):109-12.

12.	Williamson EJ, Aitken Z, Lawrie J, Dharmage SC, Burgess JA, Forbes AB. Introduction to causal diagrams for confounder selection. Respirology (Carlton, Vic). 2014;19(3):303-11.

13.	Lederer DJ, Bell SC, Branson RD, Chalmers JD, Marshall R, Maslove DM, et al. Control of Confounding and Reporting of Results in Causal Inference Studies. Guidance for Authors from Editors of Respiratory, Sleep, and Critical Care Journals. Annals of the American Thoracic Society. 2019;16(1):22-8.

14.	Tennant PWG, Murray EJ, Arnold KF, Berrie L, Fox MP, Gadd SC, et al. Use of directed acyclic graphs (DAGs) to identify confounders in applied health research: review and recommendations. International journal of epidemiology. 2021;50(2):620-32.
